# Enhanced External Counterpulsation Intervention Induces the Variation of Physiological Parameters and Shear Stress Metrics in the Carotid Artery

**DOI:** 10.3390/bioengineering12040386

**Published:** 2025-04-03

**Authors:** Zhenfeng Ren, Zi’an Wu, Yanjing Wang, Israilov Jakhongirkhon, Qianxiang Zhou, Jianhang Du

**Affiliations:** 1 Key Laboratory of Biomechanics and Mechanobiology (Beihang University), Ministry of Education, Key Laboratory of Innovation and Transformation of Advanced Medical Devices, Ministry of Industry and Information Technology, National Medical Innovation Platform for Industry-Education Integration in Advanced Medical Devices (Interdiscipline of Medicine and Engineering), School of Biological Science and Medical Engineering, Beihang University, Beijing, 100191, China; by2110132@buaa.edu.cn (Z.R.); by1610124@buaa.edu.cn (Y.W.); 2 Medical Research Center, The Eighth Affiliated Hospital of Sun Yat-sen University, Shenzhen 518033, China; wuzhzh27@mail2.sysu.edu.cn; 3 National Health Commission (NHC) Key Laboratory of Assisted Circulation, Sun Yat-sen University, Guangzhou 510080, China; 4 Department of Public Health and Administration, Tashkent Medical Academy, Tashkent 100109, Uzbekistan; j.d.israilov@gmail.com

**Keywords:** atherosclerotic lesion, hemodynamic, external counterpulsation, wall shear stress, oscillatory

## Abstract

Enhanced external counterpulsation (EECP) treatment has been demonstrated to be effectively vasculoprotective and anti-atherosclerotic in clinical observations and controlled trials. The diastolic blood flow augmentation induced by EECP greatly affected the local hemodynamic environment in multiple arterial segments. In this study, a porcine model of hypercholesterolaemia was developed to perform an invasive physiological measurement involving electrocardiogram, blood flow wave, and arterial pressure. Subsequently, a three-dimensional carotid bifurcation model was developed to evaluate the variations in wall shear stress (WSS) and its temporal and spatial oscillations. The results show that, compared to the pre-EECP state, EECP stimulus led to an increase of 28.7% in the common carotid artery (CCA) blood flow volume over a cardiac cycle, as well as an augmentation of 22.73% in the diastolic pressure. Meanwhile, the time-average wall shear stress (TAWSS) over the cardiac cycle increased 25.1%, while the relative residence time (RRT) declined 45.7%. These results may serve to reveal the hemodynamic mechanism of EECP treatment that contributes to its anti-atherosclerotic effects.

## 1. Introduction

**Figure 1:**
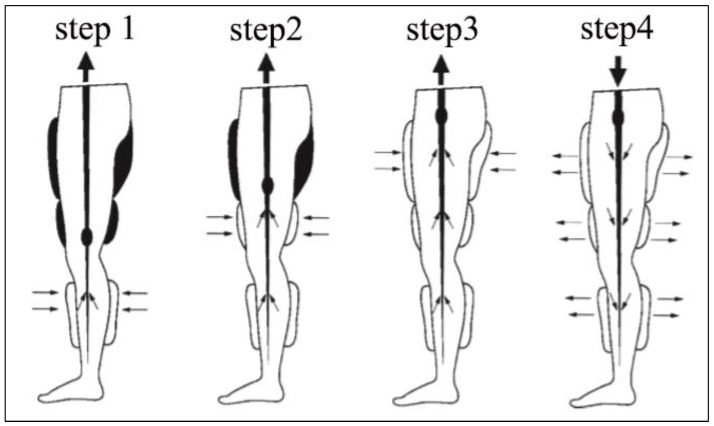
Technique of EECP treatment. It contains sequential inflations and deflations according to the electrocardiosignal. During diastole, the cuffs are sequentially inflated, from distal to proximal. Three steps are involved, including calves (step1) to lower thighs (step2) and then to upper thighs (step3). In turn, deflation (step4) is performed at the onset of systole, resulting in the release of pressure.

Enhanced external counterpulsation (EECP), which was originally derived from our laboratory, is a U.S. Food and Drug Administration (FDA)-approved non-invasive technology for the improvement and augmentation of hemodynamics and blood perfusion in ischemic organs and tissue [1,2,3,4,5,6,7]. The EECP technique (see Figure 1) involves the sequential inflations and deflations of pneumatic cuffs wrapped around the patient’s hip and lower limbs, which is triggered by the electrocardiosignal [1]. As the result, EECP stimulus can lead to multiple acute hemodynamic benefits [8] including diastolic augmentation similar to intra-aortic balloon pump (IABP), venous return augmentation and afterload reduction.

Except for the acute hemodynamic effects, long-term EECP treatment was demonstrated for the potential anti-atherosclerosis lesion effect via the improvement of endothelial functions, with an underlying mechanism similar to physical exercise [9]. Studies based on human subjects and animal models confirmed that one 35 h course of EECP treatment could produce following advantages: (I) significant augmentation of reactive hyperemia-peripheral arterial tonometry (RH-PAT) [10]; (II) significant improvement of hypercholesterolemia-induced diminished endothelium-dependent vasorelaxation [11]; (III) decrease in arterial wall stiffness and (IV) reduce in myocardial oxygen demand [12]. Previous studies of our group confirmed that EECP could inhibit intimal hyperplasia by modifying shear stress-responsive gene expression in hypercholesterolemic porcine models [9]. EECP-induced wall shear stress (WSS) effects were generally believed to play a crucial role in explaining its subsequent vasculoprotective effects [9,13]. However, the proper effects of EECP stimulus on WSS and its temporal and spatial oscillation have not been fully clarified yet, especially in atherosclerotic lesion-prone sites.

It is widely believed that low and/or oscillating WSS is in positive correlation with the generation and development of atherosclerotic lesion [14,15,16]. To represent the magnitude and fluctuation of WSS over one cardiac cycle, WSS-derived hemodynamic metrics such as time average WSS (TAWSS), oscillatory shear index (OSI), relative resident time (RRT), and wall shear stress gradient (WSSG) were proposed and regarded as potential biomechanical indicators for the assessment of atherosclerotic risk [15,17,18,19].

The current paper aimed to present a combined experimental and numerical study to evaluate the influence of EECP intervention on local hemodynamic environment in carotid artery bifurcation. The WSS metrics were numerically solved in both conditions of pre-EECP (at rest) and during EECP intervention and, in turn, were compared. The current study was supposed to contribute to explaining the hemodynamic mechanism underlying the vasculoprotective and anti-atherosclerotic effects of long-term EECP treatment.

## 2. Animal Experiment

### 2.1. Animal Model

One male crossbred (Yorkshire (male) and Landrance (female)) porcine model (110 days of age and 55.2 kg of weight) with high-fat-feeding-induced hypercholesterolaemia was enrolled in this study. All procedures were performed following the Guide for the Care and Use of Laboratory Animals published by the US National Institutes of Health. The subjects was anesthetized with midazolam 5 to 10 mg IM and an intraperitoneal injection of 3% pentobarbital 10 mg/kg per h [9]. This study was approved by the local medical ethics committee of the Eighth Affiliated Hospital of Sun Yat-sen University (No. 2024-140-01).

### 2.2. EECP Intervention

The experimental subject received a 45 min single session of EECP treatment based on an EECP device system (EECP-MCI, Shuangshan, Foshan, China) equipped with a platform and cuffs specially designed for a porcine model. The subject was laid on its left side. The working pressure of the cuffs was set at 35 kPa to guarantee the effective augmentation of diastolic pressure during EECP (diastolic-to-systolic ratio ≥ 1.2) by monitoring the finger pulse based on the plethysmographic technique.

### 2.3. Invasive Physiological Measurement

Blood flow rate waveforms in the cardiac cycles were measured in the right carotid artery, which was exposed surgically, by using of an electromagnetic flowmeter (MFV-3200, Nihon Kohden, Tokyo, Japan) equipped with a 4–5 mm probe. Meanwhile, the left carotid artery was cannulated based on the Seldinger technique to in turn conduct a blood pressure monitoring using tip pressure transducer (TP-400T, Nihon Kohden, Tokyo, Japan). Electrocardiogram (ECG) readings were detected based on a standard three-lead module. All the measuring data were synchronized and recorded via a multi-channel physiological recorder (MP150CE, BIOPAC, Goleta, CA, USA).

**Figure 2:**
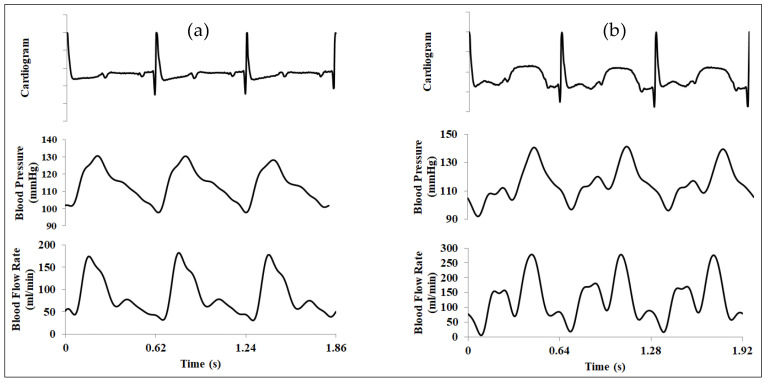
In vivo hemodynamic measurements in the CCA. (**a**) pre-EECP; (**b**) during EECP. Note that blood flow rate, as well as blood pressure, was suppressed by EECP during systole; however, it was apparently enhanced during diastole.

The physiological measurements pre- and during EECP in three continuous cardiac cycles are graphically compared in Figure 2. EECP stimulus induced apparent variations in both blood flow pattern and blood pressure waveform in CCA. Mean CCA blood flow volume over a cardiac cycle exhibited an increase of 35.24% during EECP treatment compared with the pre-EECP condition, as well as an augmentation of 22.17% in the diastolic pressure.

## 3. Numerical Simulation Procedure

### 3.1. Geometry Reconstructions and Boundary Condition Settings

**Figure 3:**
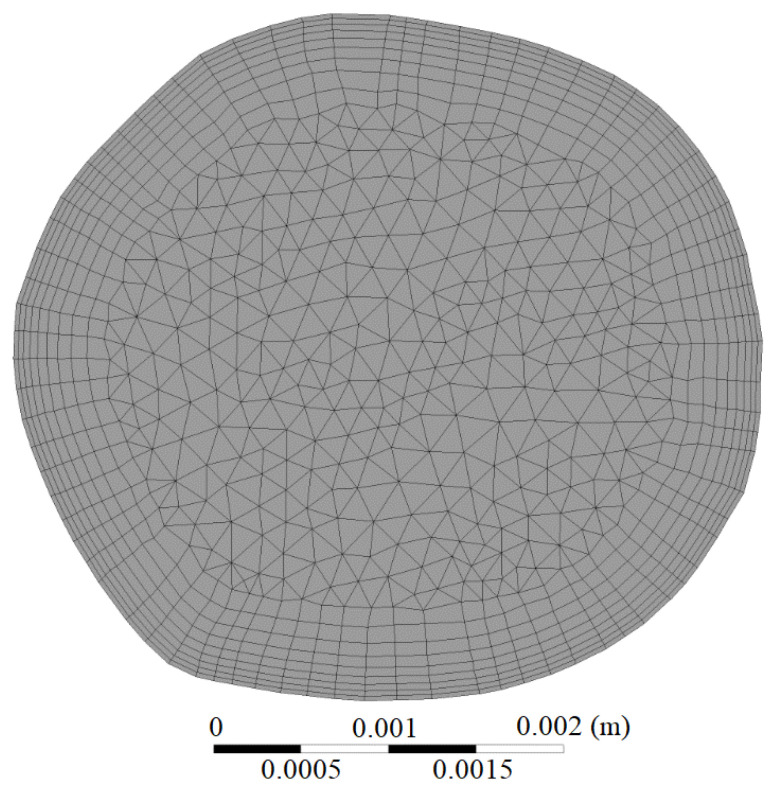
Schematic mesh at the inlet.

Three-dimensional geometries of the carotid artery bifurcation were constructed based on magnetic resonance angiography (MRA) images and the commercial package of MIMICS 17.0. The vessel wall was considered to be rigid in the understanding of flow behavior pre- and during EECP stimulus. As shown in Figure 3, the original geometry involved four boundaries: an inlet, two outlets, and a wall. The inflow rate, measured by electromagnetic flowmeter at CCA, was used for the boundary condition of inlet_ex, which was achieved by extending the inlet boundary outward along its normal direction for a length of 5 times its diameter. This could guarantee a fully developed velocity profile at the inlet boundary. Free boundary conditions were applied at the outlets. A no-slip condition was set at the wall.

### 3.2. Mesh Generation and Mesh Independence Analysis

Mesh generation was achieved with package of ICEM 17.0. Mesh with tetrahedral cells was performed in the core domain of the geometry. A boundary layer with 9 inflation layers were generated at the near-wall region to improve solution accuracy where high variable gradient exists (see Figure 3). The mesh independence analysis was performed to obtain an optimized meshing strategy for both reliable numerical results and acceptable computational burdens. Table 1 shows the dependence of TAWSS on the mesh size, a convergent value was expected to be obtained as the mesh density increases. The final mesh used in this study contained a quantity of about 1.7 million elements.

### 3.3. Governing Equations and the Solver

The Newtonian fluid assumption of blood was adopted in the current study, which had been widely recommended to be suitable for large and medium arteries [20]. The incompressible and isoviscous blood fluid transportation in the artery is governed by the normal Navier–Stokes equations.

The finite-volume-method-based CFX software (18.2 package, Ansys Inc., Canonsburg, PA, USA) was used to discretize and solve the governing equations. Reliable numerical solutions were achieved only when the root mean square of both mass and momentum residuals was equal to or less than 10^−5^. A simulation involving two cardiac cycles was needed in this case with respect to this configuration. The numerical results presented below were extracted from the simulation of the second cardiac cycle.

### 3.4. Wall Shear-Stress-Derived Hemodynamic Metrics

To start the analysis of results, it is necessary to provide the definitions of those WSS-derived hemodynamic metrics as [6,17].

(1) $$\mathrm{TAWSS}=\frac{1}{T}\int_{0}^{T}\left|{\vec{\tau }}_{w}\right|dt$$

(2) $$\mathrm{WSSG}=\sqrt{(\left|\frac{\partial {\vec{\tau }}_{w}}{\partial x}\right|{)}^{2}+(\left|\frac{\partial {\vec{\tau }}_{w}}{\partial y}\right|{)}^{2}+(\left|\frac{\partial {\vec{\tau }}_{w}}{\partial z}\right|{)}^{2}}$$

(3) $$\mathrm{AWSSG}=\frac{1}{T}\int_{0}^{T}\mathrm{WSSG}dt$$

(4) $$\mathrm{OSI}=\frac{1}{2}(1-\frac{\left|\int_{0}^{T}{\vec{\tau }}_{w}dt\right|}{\int_{0}^{T}\left|{\vec{\tau }}_{w}\right|dt})$$

(5) $$\mathrm{RRT}=\frac{1}{(1-2\mathrm{OSI})\times \mathrm{TAWSS}}$$

where $\left|{\vec{\tau }}_{w}\right|$ is the magnitude of the instantaneous WSS vector ${\vec{\tau }}_{w}$ and *T* is the duration of one cardiac cycle.

## 4. Computational Results and Discussion

### 4.1. The Influence of EECP on WSS Distribution

WSS is the tangential frictional force exerted by blood flow on the vascular surface. Over the last few decades, a large body of publications have demonstrated that WSS is a key instigator for endothelial dysfunction and plaque process [14,15,16]. The high and positive correlation between atherosclerotic lesion and regions of low WSS has been well established, as well as the anti-atherosclerosis effect of high WSS distribution up to a certain threshold.

**Figure 4:**
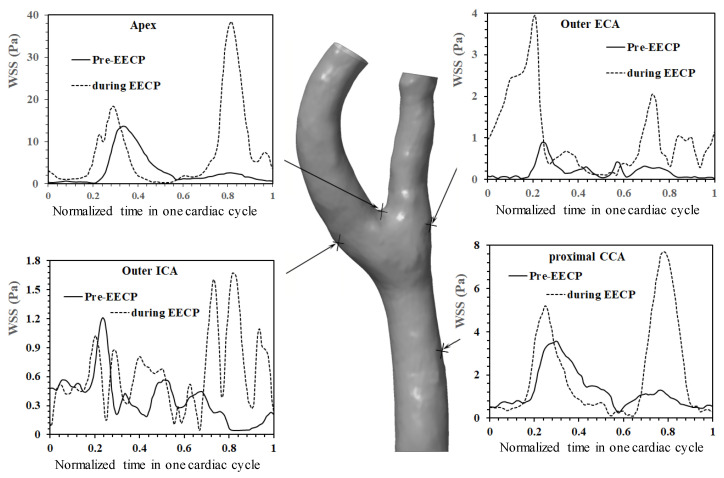
WSS distributions at different sites of carotid bifurcation in a cardiac cycle, and the comparison between the pre-EECP and during EECP conditions.

Figure 4 shows the WSS distributions at four different spatial positions, namely, the proximal CCA, outer ICA, outer ECA, and apex of the bifurcation. The impact of EECP stimulus caused the apparent variations in the WSS waveforms, especially in the diastole, as well as the promotion of WSS level. The peak WSS value in the cardiac cycle is found at the apex (13.24 Pa of Pre-EECP state at *t* = 0.336T versus 38.90 Pa of during EECP state at *t* = 0.816T).

### 4.2. The Effects of EECP Stimulus on WSS Metrics

Considering the continuous and oscillatory characteristics of WSS, TAWSS was proposed as a WSS metric to represent the average WSS magnitude over one cardiac cycle. Previous studies confirmed the high sensitivity of TAWSS in predicting plaque locations [18]. It was suggested that the increase in atherosclerotic plaque area was associated with low-TAWSS (<1.0 Pa) arterial segments, while intermediate (≥1.0 and <2.5 Pa) and high TAWSS (≥2.5 Pa) led to the decrease in plaque area [21].

**Figure 5:**
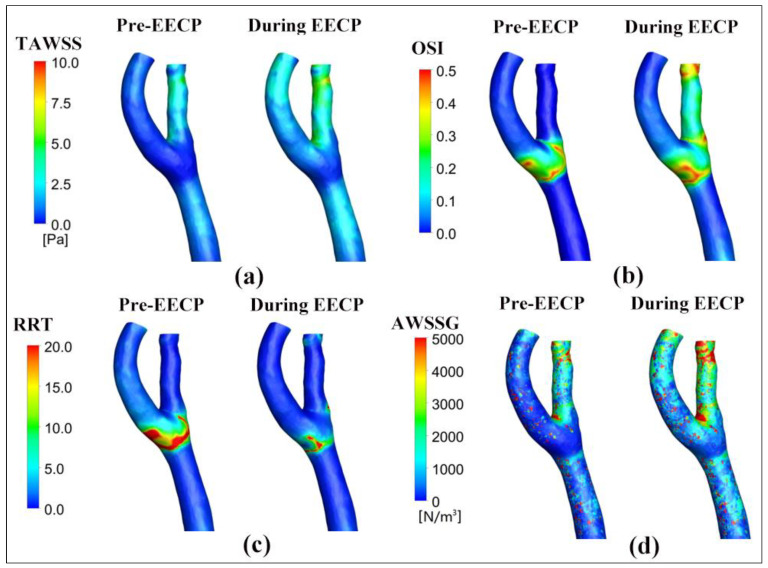
The distributions of TAWSS (**a**), OSI (**b**), RRT (**c**), and AWSSG (**d**) over one cardiac cycle under both pre-EECP and during EECP conditions.

**Figure 6:**
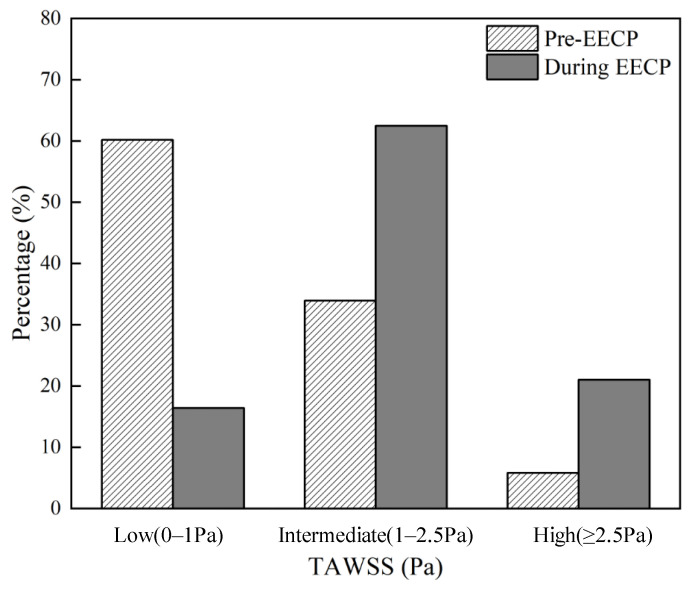
Percentages of area occupation with different bands of TAWSS.

Contours of the TAWSS pre- and during EECP stimulus are graphically compared in Figure 5a. The EECP intervention markedly elevated TAWSS throughout the bifurcation region, with a consequent 88.8% increase in the area-averaged TAWSS value. The percentages of area occupation on the carotid bifurcation wall with different bands of TAWSS level were calculated as well and are shown in Figure 6. The percentage of low TAWSS was decreased from 60.20% to 16.45% by the effect of EECP intervention, while the percentage of high TAWSS was increased from 5.86% to 21.04%. Peak values of TAWSS are found at the distal of the ECA (8.5 Pa of during EECP versus 5.7 Pa of Pre-EECP). The results are consistent with our previous study on patients with neurological disorders [8].

Contours of the OSI pre- and during EECP stimulus are graphically compared in Figure 5b. OSI was proposed to quantitatively assess the temporal fluctuation of WSS over the cardiac cycle, and demonstrated, in some previous studies, a positive spatial correlation with sites of atherosclerotic plaque formation [18,22]. Our results indicated that EECP stimulus apparently augmented the OSI level in proximal CCA and ECA and led to a 173.1% augmentation over the entire carotid artery bifurcation. However, in our previous study, we found that EECP led to a plateau or a slight augmentation of OSI in both healthy individuals and patients with neurological disorders [8].

Contours of the RRT pre- and during EECP stimulus were graphically compared in Figure 5c. RRT is a WSS metric proposed to quantitatively assess the residence time of the particles and large molecules spent in vasculature over one cardiac cycle. The value of RRT relies on the values of both TAWSS and OSI. Previous studies confirmed that the generation of atherosclerotic lesion was positively correlated with local high level RRT distribution [18,23]. Our computational results indicated that EECP intervention apparently reduces the RRT level, especially in the bold and ECA, and led to a 51.61% decrease over the entire bifurcation region.

Contours of the AWSSG pre- and during EECP stimulus were graphically compared in Figure 5d. WSSG is a WSS metric proposed to quantitatively assess the spatial fluctuation of WSS in one cardiac cycle, and AWSSG represents the time-average magnitude of WSSG over the cardiac cycle. A high WSSG level was demonstrated in some studies as having a possible correlation with neointimal hyperplasia [17,19,24]. Our computational results indicated that EECP intervention augmented the AWSSG level over the bifurcation region and especially in ICA.

## 5. Conclusions

The current paper conducted a study to assess the impact of EECP stimulus on physiological parameters and WSS-derived hemodynamic metrics in carotid bifurcation. Our results demonstrated that EECP induced an apparent augmentation in blood perfusion as well as TAWSS, while inducing a decrease in the percentage of low TAWSS area occupation as well as RRT level. The increase in WSS level induced by EECP was very close to that induced by moderate physical exercise and might fall into the vasculoprotective range [25].

We thus suggested that the positive effects of EECP treatment on TAWSS and RRT might partially contribute to the subsequent anti-atherosclerotic effects. However, EECP intervention increased the OSI and AWSSG level in carotid bifurcation at the same time; further study may need to be conducted to evaluate the possible influence.

## Figures and Tables

**Table 1:** Numerical results comparison of TAWSS with different mesh quantities.

**Mesh Quantity**		444,236	800,064	1,728,230	2,996,975
**TAWSS (Pa)**	**Pre EECP**	8.549	8.267	8.023	8.070
	**During EEECP**	8.650	8.504	8.425	8.490
**Enhancement (-)**		1.012	1.029	1.050	1.052

## Data Availability

The data presented in this study are available on request from the corresponding author.

## References

[1] Zheng Z., Yu L., Cai S., Kambic H., Li T., Ma H., Chen P., Huang B., Nosé Y. (1984). New Sequential External Counterpulsation for the Treatment of Acute Myocardial Infarction. Artif. Organs.

[2] Werner D., Schneider M., Weise M., Nonnast-Daniel B., Daniel W.G. (1999). Pneumatic External Counterpulsation: A New Noninvasive Method to Improve Organ Perfusion. Am. J. Cardiol..

[3] Michaels A.D., Accad M., Ports T.A., Grossman W. (2002). Left Ventricular Systolic Unloading and Augmentation of Intracoronary Pressure and Doppler Flow during Enhanced External Counterpulsation. Circulation.

[4] Braith R.W., Conti C.R., Nichols W.W., Choi C.Y., Khuddus M.A., Beck D.T., Casey D.P. (2010). Enhanced External Counterpulsation Improves Peripheral Artery Flow-Mediated Dilation in Patients with Chronic Angina: A Randomized Sham-Controlled Study. Circulation.

[5] Lin W., Xiong L., Han J., Leung T.W.H., Soo Y.O.Y., Chen X., Wong K.S.L. (2012). External Counterpulsation Augments Blood Pressure and Cerebral Flow Velocities in Ischemic Stroke Patients with Cerebral Intracranial Large Artery Occlusive Disease. Stroke.

[6] Qin X., Deng Y., Wu D., Yu L., Huang R. (2016). Does Enhanced External Counterpulsation (EECP) Significantly Affect Myocardial Perfusion?: A Systematic Review & Meta-Analysis. PLoS ONE.

[7] Du J., Peng J., Shen X., Li X., Zhong H., Gao Z., Chen M., Qi L., Xie Q. (2023). Enhanced External Counterpulsation Treatment Regulates Blood Flow and Wall Shear Stress Metrics in Femoral Artery: An in Vivo Study in Healthy Subjects. J. Biomech..

[8] Tian S., Pan W., Peng J., Wang H., Deng B., Liang Y., Li X., Liu H., Wang Y., Luo B. (2021). Hemodynamic Responses in Carotid Bifurcation Induced by Enhanced External Counterpulsation Stimulation in Healthy Controls and Patients with Neurological Disorders. Front. Physiol..

[9] Zhang Y., He X., Chen X., Ma H., Liu D., Luo J., Du Z., Jin Y., Xiong Y., He J. (2007). Enhanced External Counterpulsation Inhibits Intimal Hyperplasia by Modifying Shear Stress–Responsive Gene Expression in Hypercholesterolemic Pigs. Circulation.

[10] Bonetti P.O., Barsness G.W., Keelan P.C., Schnell T.I., Pumper G.M., Kuvin J.T., Schnall R.P., Holmes D.R., Higano S.T., Lerman A. (2003). Enhanced External Counterpulsation Improves Endothelial Function in Patients with Symptomatic Coronary Artery Disease. J. Am. Coll. Cardiol..

[11] Tao J., Tu C., Yang Z., Zhang Y., Chung X.-L., Ma H., Zhen Z.-S. (2006). Enhanced External Counterpulsation Improves Endothelium-Dependent Vasorelaxation in the Carotid Arteries of Hypercholesterolemic Pigs. Int. J. Cardiol..

[12] Casey D.P., Beck D.T., Nichols W.W., Conti C.R., Choi C.Y., Khuddus M.A., Braith R.W. (2011). Effects of Enhanced External Counterpulsation on Arterial Stiffness and Myocardial Oxygen Demand in Patients with Chronic Angina Pectoris. Am. J. Cardiol..

[13] Raza A., Steinberg K., Tartaglia J., Frishman W.H., Gupta T. (2017). Enhanced External Counterpulsation Therapy: Past, Present, and Future. Cardiol. Rev..

[14] Brown A.J., Teng Z., Evans P.C., Gillard J.H., Samady H., Bennett M.R. (2016). Role of Biomechanical Forces in the Natural History of Coronary Atherosclerosis. Nat. Rev. Cardiol..

[15] Mohamied Y., Rowland E.M., Bailey E.L., Sherwin S.J., Schwartz M.A., Weinberg P.D. (2015). Change of Direction in the Biomechanics of Atherosclerosis. Ann. Biomed. Eng..

[16] Thondapu V., Bourantas C.V., Foin N., Jang I.-K., Serruys P.W., Barlis P. (2017). Biomechanical Stress in Coronary Atherosclerosis: Emerging Insights from Computational Modelling. Eur. Heart J..

[17] Buchanan J.R., Kleinstreuer C., Truskey G.A., Lei M. (1999). Relation between Non-Uniform Hemodynamics and Sites of Altered Permeability and Lesion Growth at the Rabbit Aorto-Celiac Junction. Atherosclerosis.

[18] Rikhtegar F., Knight J.A., Olgac U., Saur S.C., Poulikakos D., Marshall W., Cattin P.C., Alkadhi H., Kurtcuoglu V. (2012). Choosing the Optimal Wall Shear Parameter for the Prediction of Plaque Location—A Patient-Specific Computational Study in Human Left Coronary Arteries. Atherosclerosis.

[19] Murphy J., Boyle F. (2010). Predicting Neointimal Hyperplasia in Stented Arteries Using Time-Dependant Computational Fluid Dynamics: A Review. Comput. Biol. Med..

[20] Iasiello M., Vafai K., Andreozzi A., Bianco N. (2017). Analysis of Non-Newtonian Effects within an Aorta-Iliac Bifurcation Region. J. Biomech..

[21] Samady H., Eshtehardi P., McDaniel M.C., Suo J., Dhawan S.S., Maynard C., Timmins L.H., Quyyumi A.A., Giddens D.P. (2011). Coronary Artery Wall Shear Stress Is Associated with Progression and Transformation of Atherosclerotic Plaque and Arterial Remodeling in Patients with Coronary Artery Disease. Circulation.

[22] Ku D.N., Giddens D.P., Zarins C.K., Glagov S. (1985). Pulsatile Flow and Atherosclerosis in the Human Carotid Bifurcation. Positive Correlation between Plaque Location and Low Oscillating Shear Stress. Arterioscler. Off. J. Am. Heart Assoc. Inc..

[23] Hoi Y., Zhou Y.-Q., Zhang X., Henkelman R.M., Steinman D.A. (2011). Correlation between Local Hemodynamics and Lesion Distribution in a Novel Aortic Regurgitation Murine Model of Atherosclerosis. Ann. Biomed. Eng..

[24] LaDisa J.F., Olson L.E., Douglas H.A., Warltier D.C., Kersten J.R., Pagel P.S. (2006). Alterations in Regional Vascular Geometry Produced by Theoretical Stent Implantation Influence Distributions of Wall Shear Stress: Analysis of a Curved Coronary Artery Using 3D Computational Fluid Dynamics Modeling. Biomed. Eng. Online.

[25] Green D.J., Smith K.J. (2018). Effects of Exercise on Vascular Function, Structure, and Health in Humans. Cold Spring Harb. Perspect. Med..

